# Sex-specific genetic association between psychiatric disorders and cognition, behavior and brain imaging in children and adults

**DOI:** 10.1038/s41398-022-02041-6

**Published:** 2022-08-26

**Authors:** Yuanyuan Gui, Xiaocheng Zhou, Zixin Wang, Yiliang Zhang, Zhaobin Wang, Geyu Zhou, Yize Zhao, Manhua Liu, Hui Lu, Hongyu Zhao

**Affiliations:** 1 State Key Laboratory of Microbial metabolism, Joint International Research Laboratory of Metabolic & Developmental Sciences, Department of Bioinformatics and Biostatistics, School of Life Sciences and Biotechnology, Shanghai Jiao Tong University, Shanghai, China; 2 SJTU-Yale Joint Center for Biostatistics and Data Science, Department of Bioinformatics and Biostatistics, School of Life Sciences and Biotechnology, Shanghai Jiao Tong University, Shanghai, China; 3 Department of Biostatistics, Yale School of Public Health, New Haven, CT USA; 4 Program in Computational Biology and Bioinformatics, Yale University, New Haven, CT USA; 5 MoE Key Laboratory of Artificial Intelligence, AI Institute, Shanghai Jiao Tong University, Shanghai, China

**Keywords:** Psychiatric disorders, Genetics

## Abstract

Although there are pronounced sex differences for psychiatric disorders, relatively little has been published on the heterogeneity of sex-specific genetic effects for these traits until very recently for adults. Much less is known about children because most psychiatric disorders will not manifest until later in life and existing studies for children on psychiatric traits such as cognitive functions are underpowered. We used results from publicly available genome-wide association studies for six psychiatric disorders and individual-level data from the Adolescent Brain Cognitive Development (ABCD) study and the UK Biobank (UKB) study to evaluate the associations between the predicted polygenic risk scores (PRS) of these six disorders and observed cognitive functions, behavioral and brain imaging traits. We further investigated the mediation effects of the brain structure and function, which showed heterogeneity between males and females on the correlation between genetic risk of schizophrenia and fluid intelligence. There was significant heterogeneity in genetic associations between the cognitive traits and psychiatric disorders between sexes. Specifically, the PRSs of schizophrenia of boys showed stronger correlation with eight of the ten cognitive functions in the ABCD data set; whereas the PRSs of autism of females showed a stronger correlation with fluid intelligence in the UKB data set. Besides cognitive traits, we also found significant sexual heterogeneity in genetic associations between psychiatric disorders and behavior and brain imaging. These results demonstrate the underlying early etiology of psychiatric disease and reveal a shared and unique genetic basis between the disorders and cognition traits involved in brain functions between the sexes.

## Introduction

Schizophrenia (SCZ), bipolar disorder (BD), major depressive disorder (MDD), and autism spectrum disorder (ASD) are common, highly heritable, and sex-specific psychiatric disorders that are related to cognitive function impairments, psychiatric behaviors anomalies, and brain abnormalities [1–5], which are also highly heritable and sex-specific. The lifetime prevalence of SCZ, BD, MDD, and ASD is about 1, 0.4–4.0, 20, and 0.76%, respectively, and their heritability is approximately 79, 70, 35, and 90%, respectively [6–9]. In addition, hundreds of genomic regions were shared by these neuropsychiatric traits, and tissue-specific functional annotation results showed that the genomic regions are significantly enriched in brain [10]. The sex differences of psychiatric disorders are reflected in the age of onset, symptoms, brain morphology and functions, and cognitive and behavioral dysfunctions [1–4, 11].

SCZ is characterized by three core features: positive symptoms, negative symptoms, and cognitive impairment [12]. The first episode of SCZ usually occurs in late adolescence or early adulthood. There is usually a prodromal phase with an average duration of 5 years [13, 14]. In some cases, premorbid impairment in cognition and/or social functions dates back many years [15]. Some literature demonstrates differences in age of onset distributions between males and females. Specifically, it was reported that males usually have onset 3–4 years earlier than females and show a rapid increase around 14–21 years old. In addition, males with SCZ tend to have more serious cognitive deficits [16]. The explanations of the sex differences in cognitive cognitions are attributed to sex differences in brain [1, 17].

BD is a complex disorder characterized by extreme fluctuations in the mood with the age of onset about 25 [18]. There are three subtypes: type 1 bipolar disorder (BD1), type 2 bipolar disorder (BD2), and schizoaffective bipolar disorder (SAB). The lifetime prevalence of BD is similar between males and females, despite some literature suggesting sex differences in the rates and clinical features of BD1 and BD2 [3]: the prevalence of BD1 or manic episodes was higher in males while the prevalence of BD2 or hypomania was higher in females. However, few studies have assessed sex-related differences in the characteristics of BD such as cognitive impairment, psychiatric behavior anomalies, and brain abnormalities.

The sex differences in MDD are age-specific. In pre-puberty, boys and girls have similar rates of depression. During adolescence and adulthood, MDD in females is more than twice as prevalent as that in males, whereas the prevalence in women is reduced to the level of men in senior age [19]. Similarly, only a few studies have assessed sex differences in cognitive function in MDD. Interestingly, inconsistent brain abnormalities have been found in adolescents and adults with depression. The sex differences in brain abnormalities are also age-dependent [20, 21]. These results suggest that MDD has a dynamic impact on brain morphology which has different patterns of alterations at different stages of life.

ASD is an early-onset disorder, with a ratio of prevalence 2–5:1 [22] of boys over girls. Although researchers have compared autism-related brain abnormalities and behavioral cognitive traits along with their heterogeneity of genetic architectures between males and females [18, 23–25], few studies have jointly investigated these issues.

Although there are pronounced sex differences for psychiatric disorders and related traits, the focus of these differences in previous studies has mainly been on phenotypic comparisons among adults. Few have been published on the heterogeneity of sex-specific genetic effects for these traits until very recently for adults [25]. Even fewer studies focus on children because most psychiatric disorders will not manifest until later in life and existing studies for children on psychiatric traits such as cognitive functions are underpowered. The correlation between psychiatric disorder genetics and cognitions in the general population is also poorly known. However, later childhood to early adolescence is a crucial developmental period for brain and neuropsychiatric traits. Although some psychiatric disorders do not appear in childhood, these disorders have a prodromal phase, including premorbid abnormalities in behavior, brain, and cognition. Those occur during childhood development characterized by a spectrum of symptoms [26–29]. Therefore, it is particularly important to study the genetic associations between mental illness and related traits for children at this critical stage of development. Furthermore, the human brain, cognition, behavior, and disease process change throughout life and are dynamically related to each other. The genetic associations between psychiatric disorders and related traits also change with human development. However, so far, no study with decent statistical power has directly compared the genetic effects of psychiatric disorders on related traits at different ages, which could facilitate developmentally-sensitive risk prediction and treatment of psychiatric disorders.

Our main intentions here were to show the sex differences in the genetic effects of psychiatric disorders on related traits, compare the sex differences between children and adults, and illustrate the underlying mechanisms. We analyzed data from two large studies, Adolescent Brain Cognitive Development (ABCD) study and UK Biobank (UKB), which offer rich information on genetics, cognitive functions, clinical and behavioral traits. We used the correlation between PRS of psychiatric disorders and related traits as the measure for genetic association. Analysis based on the general population may more likely reduce the confounding effects of medication and other secondary disease factors. The PRS of SCZ is the only one that was significantly associated with most cognitive traits in both children and adults. However, although the correlation between the PRS of SCZ and cognitive traits showed significant sex differences in children, we did not identify significant sex differences in them for adults. To investigate the mediation effects of brain imaging traits on the association between SCZ PRS and cognitions, we further performed mediation analysis on the brain structure and function which showed sexual heterogeneity.

## Materials and methods

### Study cohorts

The ABCD [30] study recruited 11,875 children aged 9–10 from 21 research sites across the United States between 2015 and 2019 to track the study participants for at least ten years. Details concerning demographic, physical, and mental health factors for these children are described in Barch et al. [31]. Our study used the second public ABCD data release (10.15154/1503209).

The UKB [32] is a prospective study of over 500,000 individuals aged 40–69 recruited in the United Kingdom from 2006 to 2010. All participants provided informed consent, and the UK Biobank’s Research Ethics Committee approved the study. Detailed project information can be found on the website (http://www.ukbiobank.ac.uk).

### Genotype data

The genotype data for ABCD comprised 10,627 samples and were released in 2019. SNPs and subjects were excluded based on the standard quality control criteria (Supplementary Materials). SHAPEIT [33] and IMPUTE2 [34] were used to phase and impute the 5735 individuals of European ancestry using the 1000 Genomes Project phase 3 [35] imputation reference panel. After filtering, the ABCD dataset retained 3,326,238 variants and 4722 individuals. More details of the quality control are provided in Fig. S1A.

For UKB imputed genotype data [36], we selected 409,605 European ancestry individuals defined by data field 22006. Of these, 32,237 individuals have 519 brain imaging-derived phenotypes (IDPs) with no missing values. 212,626 individuals have fluid intelligence scores. Additional quality control was performed in the same way described in ABCD (Supplementary Materials). Samples were identified as outliers in heterozygosity according to field 22027. Genetic kinship to other participants was defined by field 22021. The first ten genetic principal components of genotyped data were used as covariates for adjustments of population stratification.

### Measures

#### Cognitive measures

NIH Toolbox was used to measure cognition in ABCD. It consists of seven tasks and three composite scores for total cognition, fluid cognition, and crystallized cognition [37]. All scores were baseline data and uncorrected, which were not standardized. We defined the first principal component of these seven cognitive tasks as *g* factor [38], which accounts for 40% of the total variance. G factor can be used as a general measurement of cognitive function for the children in ABCD. It showed high genetic correlation (rg = 0.812, s.e. = 0.094, *p* = 6.2 × 10^−18^) with fluid test used in UKB [39].

Two fluid intelligence score subset data were selected based on field 20016 (‘touchscreen’) and field 20191 (‘web-based’) of UKB. We used the test score collected from the initial assessment visit (2006–2010) or subsequent assessments if data were missing at the initial visit. Only the touchscreen test scores were used if participants had completed both the touch-screen and the web-based tests. In total, 147,175 unrelated participants of European ancestry with both fluid intelligence scores and genotype data remaining after quality control were included in the analysis.

For both ABCD and UKB data, we performed linear regression to adjust age using cognitive functions as the dependent variable and obtained the residuals for each cognitive function. We then compared the residuals between the sexes.

#### Behavior measures

The children’s problems and competencies were rated using the parent-reported Child Behavior Checklist (CBCL), including social activities, relationships, and school functioning. The higher the score, the more serious the problem. We investigated the sex differences in the effects of psychiatric disorders on the risk of 20 psychiatric behaviors in ABCD.

#### Brain imaging

Details of the ABCD MRI data acquisition and analysis ﻿have been published previously [40]. We considered morphometric measures, diffusion tensor imaging (DTI), and resting-state fMRI in our study. Morphometric measures consist of cortical thickness, sulcal depth, area, and subcortical volume. DTI was used to calculate several microstructural tissue properties in white matter, such as fractional anisotropy (FA) and mean diffusivity (MD). Resting-state fMRI was used to assess the functional connectivity between brain regions at rest. The individuals with detected clinical referrals or any MR scanning of poor quality were censored according to the Fix Note of Release 2.0.1 by the ABCD Consortium. Individuals recorded with any missing value in brain features or co-variables were removed. The cleaned dataset retained 2966 European ancestry individuals and 1150 brain features.

Our study used IDPs generated by an image-processing pipeline developed and ran on behalf of the UKB [41]. Details of the acquisition protocols, image processing, and image data files are documented in biobank.ctsu.ox.ac.uk/crystal/docs/brain_mri.pdf. We used IDPs including total brain volume (not normalized for head size), gray matter partial volume, white matter partial volume, and subcortical structures. Spatially-specific IDPs related to the dMRI data such as FA and MD were derived in two ways: white-matter tract skeleton and probabilistic tractography. The resting-state fMRI data were from spatial-ICA with 25 dimensions. The cleaned discovery dataset retained 21,523 European ancestry individuals who were not in kinship with any other samples according to data field 22021 and 519 IDPs. More details of these measures used in our study are provided in the Supplementary Materials (Table S1).

#### PRS constructions

We used the publicly available GWAS summary datasets downloaded from the PGC website (https://www.med.unc.edu/pgc/download-results) of six psychiatric disorders (SCZ, BD, BD1, BD2, MDD, and ASD) [42–45] to construct PRSs.

Quality control was applied to each GWAS dataset following the steps in Zhou et al. [46]. We removed strand ambiguous SNPs, insertions and deletions (INDELs), and SNPs within the MHC locus of the GWASs. Then the cleaned SNPs were intersected with the HM3 SNPs of 1000G provided in the PRS-CS [47] reference panel. The mismatched SNPs of ABCD and UKB were also excluded. The number of SNPs and individuals remaining after quality control are shown in Table 1 and Table 2, respectively.

**Table 1:** Basic characteristics of six summary statistics for psychiatric disorders in this study.

Data sets	Sample size (Case/Control)	SNPs	Age	Ancestry
Schizophrenia	65,955 (33,426/32,541)	964,423	>18	European
Bipolar disorder	41,606 (20,129/21,524)	948,997	>18	European
Bipolar I	40,211 (14,879/30,992)	937,512	>18	European
Bipolar II	11,853 (3,421/22,155)	935,293	>18	European
Major depressive disorder	156,582 (59,851/113,154)	943,785	>18	European
Autism spectrum disorder	44,367 (18,382/27,969)	916,714	>18	European

For six binary diseases, an effective sample size was used $\left( {\frac{{4 \ast N_{{\rm{case}}} \ast N_{{\rm{control}}}}}{{N_{{\rm{case}}} + N_{{\rm{control}}}}}} \right).$

**Table 2:** Basic characteristics of individuals and SNPs for ABCD and UKB data sets.

Data sets	Sample size (female/male)	SNPs	Age	Ancestry
ABCD	4722 (2,198/2,524)	523,459	9.91 ± 0.62	European
UKB (Fluid score)	147,175 (78,561/68,614)	961,560	57.90 ± 8.23	European
UKB (IDPs)	21,523 (11,180/10,343)	963,157	63.84 ± 7.47	European

*ABCD* Adolescent Brain Cognitive Development; *UKB* UK Biobank.

We constructed PRS for the six psychiatric disorders for the participants in ABCD and UKB. The PRS model was trained based on the GWAS summary statistics of the six psychiatric disorders. For each of the six diseases, each minor allele was given a risk score using SDPR [46]. The PRS of each individual included in our target dataset was calculated using Plink v1.9 using the scores output by SDPR. The associations between the PRSs of these psychiatric disorders and cognitive function, psychiatric behavior, and brain measures were examined through linear regression adjusted by age, sex, and top PCs of genotype. Total brain volume and “headsize scaling factor” were also included as covariates in linear regression, for participants from ABCD and UKB, respectively, when the morphometric measure was a subcortical structure in ABCD and IDP was a raw volumetric measure in UKB. We performed three separate sets of regression for males, females, and both sexes. We evaluated the genetic effects of six psychiatric disorders on related traits by computing their corresponding partial R^2^ in different sets of regression (variance explained by PRS). We used permutation test to assess the statistical significance of the observed partial R^2^ of PRSs of the regression for each sex. The individual PRSs were randomly permuted among the study subjects 10,000 times. For each permuted data set, we calculated the partial R^2^ of the PRSs of the regression for each sex. Based on the distribution of the partial R^2^ from the permuted samples, we calculated the *p*-values and controlled the false discovery rate (FDR) at 0.05 (detailed in Supplementary materials). To assess the significance of the difference in partial R^2^ between males and females, if the observed partial R^2^ was significant for both males and females, we followed bootstrap and resampled 1000 times to obtain 1000 samples of R^2^. The statistical significance of sex difference was evaluated by Wilcoxon test (Tables S2, S3, and S4).

To confirm the robustness of our results on brain structure and function, we replicated the results on a non-overlapping UKB dataset. There were 31,698 unrelated (beyond third degree) samples of European ancestry in our UKB dataset. Among them, 21,523 samples who were not in genetic kinship to any other samples according to data field 22021 were included in the discovery dataset. The rest 10,175 unrelated samples of European ancestry were included in the replication dataset. The non-overlapping dataset retained 10,175 unrelated European ancestry individuals and 519 IDPs.

#### Mediation analysis

Mediation analysis was carried out to investigate whether sex-specific IDPs have mediation effects on the link between SCZ PRSs and cognitive function, and their heterogeneity between the sexes. We refer to Baron et al. [48] for an introduction to mediation analysis. The mediation model is summarized in Fig. S4.

Since we assumed the individuals were independent, bootstrap was used to compute confidence intervals and test the significance of the mediation effect. We also calculated the R^2^ of the direct effect and indirect effect of SCZ PRSs on cognition in males and females and tested their sex difference using bootstrap and Wilcoxon test (detailed in Supplementary Materials).

## Results

### Sex differences of cognitive functions in children and adults

**Fig. 1:**
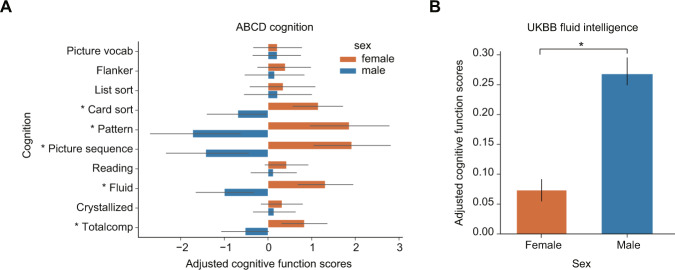
Sex differences of cognitions in healthy children and adults. The average adjusted cognitive function scores for children and adults. **A**, **B** Comparison of cognitive functions for males (blue) and females (red) in ABCD and UKB, respectively. Linear regression models were computed with cognition as the outcome and age as the predictor then the residuals were saved as adjusted cognitive function score. The error bar represents the standard deviation of adjusted scores. * <0.001.

For children in the ABCD study, girls showed higher cognitive scores than boys in five cognitive tasks (Fig. 1A), including card sort (FDR_bh_ = 9.43 × 10^−6^, effect size(*r*) = 0.068), pattern (FDR_bh_ = 8.34 × 10^−8^, *r* = 0.083), picture sequence (FDR_bh_ = 8.34 × 10^−8^, *r* = 0.083), fluid (FDR_bh_ = 3.19 × 10^−6^, *r* = 0.072), and total cognition (FDR_bh_ = 6.16 × 10^−3^, *r* = 0.043). For adults in UKB, males showed higher average fluid intelligence scores than females (*p* = 2.47 × 10^−87^, *r* = 0.052, Fig. 1B).

### Sex-specific effects of psychiatric disorder PRSs on cognitive functions

**Fig. 2:**
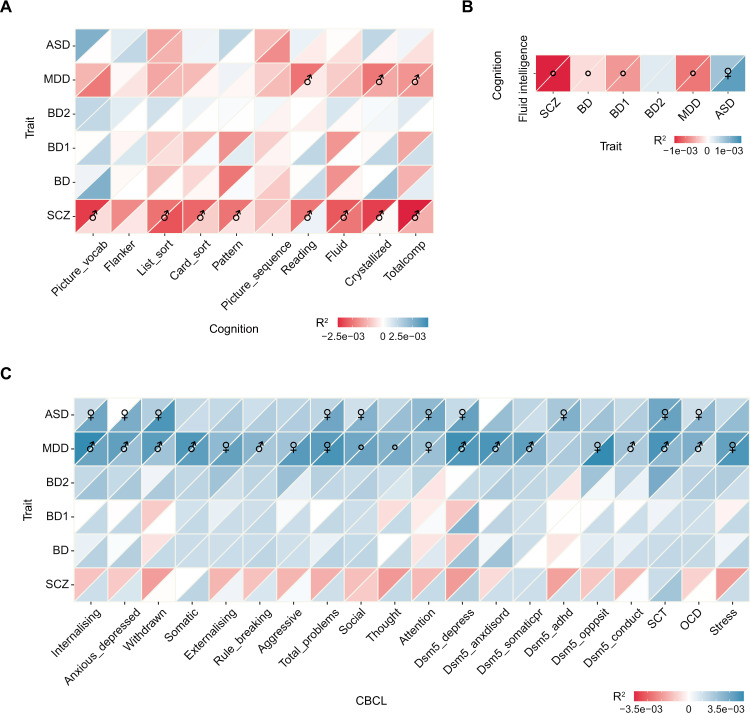
Sex-specific PRS effects for psychiatric disorders on cognitive functions and psychiatric behaviors. **A**, **B** Heat map showing effects of psychiatric disorder PRSs on cognitive functions in children and adults. **C** The effects of psychiatric disorder PRSs on psychiatric behaviors in children. The upper triangle indicates the observed partial R^2^ for males, and the lower triangle indicates the observed partial R^2^ for females. ♂ shows that the observed partial R^2^ of males was significantly higher than that of the females, and ♀ represents that females have a higher partial R^2^. ○ shows the observed partial R^2^ was significant, but there was no significant sex difference. Blue represents phenotypic positive correlation, and red represents negative correlation. *SCZ* schizophrenia, *BD* bipolar disorder, *BD1* type 1 bipolar disorder, *BD2* type 2 bipolar disorder, *MDD* major depressive disorder, *ASD* autism spectrum disorder.

We next studied the effects of PRSs of the six psychiatric disorders on cognitive functions in children and adults. We did not detect any statistically significant genetic association between the PRSs of BD, BD1, BD2, and ASD and cognitions (Table S2). PRSs of SCZ and MDD showed stronger genetic effects on cognitive functions in boys than girls. Eight out of the ten cognitive functions for boys were significantly associated with SCZ PRSs, while no significant effect of SCZ PRSs on any cognitive function was identified for girls. Similarly, PRSs of MDD did not show statistically significant effects on any of the cognitive functions in girls, whereas three out of ten cognitions (reading, crystallized, and total cognition) in boys were identified to significantly correlate with PRSs of MDD (Fig. 2A).

The PRSs of five psychiatric disorders showed significant effects on fluid intelligence both in males and females from UKB, except for BD2. In adults, genetic risks of SCZ, BD, BD1, and MDD were significantly negatively correlated with fluid intelligence with no significant sex differences identified. Interestingly, PRSs of ASD had significant positive associations with fluid intelligence in the dataset with both sexes. In terms of sex differences, females showed significantly stronger associations than males (*p* < 2.20 × 10^−16^, *r* = 0.87). There was no evidence of the association between PRSs of BD2 and fluid intelligence in UKB (*p* = 0.30–0.51, Fig. 2B, Table S3).

### Sex-specific effects of psychiatric disorder PRSs on psychiatric behaviors in children

PRSs of ASD and MDD showed significant sex-differentiated genetic effects on behaviors in ABCD. The ASD PRSs were significantly positively associated with half of the psychiatric behaviors (10 out of 20) in girls. There was no significant correlation between ASD PRSs and any behavior in boys. The MDD PRSs were significantly positively correlated with most behaviors (19 out of 20 in boys and 16 out of 20 in girls). 17 of the 20 psychiatric behaviors showed significant sex differences. To be specific, six behaviors were more strongly correlated with MDD PRSs including external, aggressive, total problem, attention, opposite, and stress in girls (*p* = 2.48 × 10^−223^–3.95 × 10^−2^, r = 0.065–1.0) and 11 behaviors had stronger associations with MDD PRSs including rule-breaking, withdrawn, etc. in boys (*p* = 5.58 × 10^−132^–1.62 × 10^−30^, *r* = 0.18–0.88). There were no significant sex differences in the effects of PRSs for SCZ, BD, BD1, and BD2 on the psychiatric behaviors in UKB (Fig. 2C and Table S2).

### Sex-specific effects of psychiatric disorder PRSs on the brain structure and function in children and adults

We studied the sex differences in the effects of psychiatric disorder PRSs on brain structure and function in ABCD and UKB (Table S5). However, neither girls’ nor boys’ PRSs of the six psychiatric disorders was identified to have significant effects on cortical and subcortical structures and rsfMRI functions in ABCD.

**Fig. 3:**
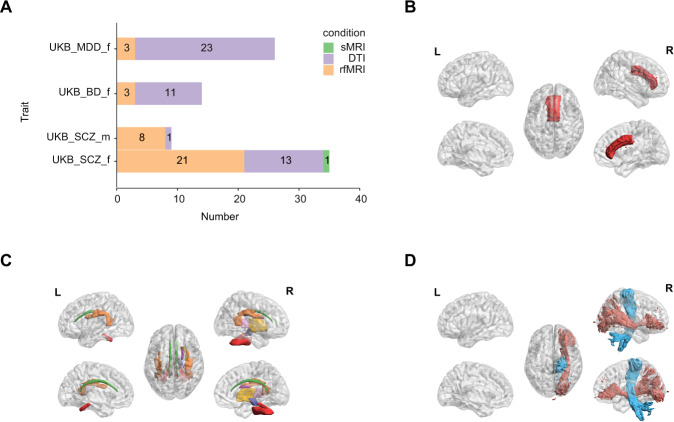
Sex differences in the effects of psychiatric disorder PRSs on the brain. The IDPs (imaging-derived phenotypes) that was significantly associated with psychiatric disorders PRSs and the association was different between sexes, referred to as sex-specific IDPs. **A** The *x*-axis shows the number of sex-specific IDPs, indicating the psychiatric disorders and the corresponding sex of stronger association (e.g., UKB_SCZ_m represents the genetic association between SCZ PRSs and brain is significantly stronger in males than females in the UKB). Different colors represent different brain imaging modalities. Orange bins and instance counts from the histogram indicate the sex-specific rsfMRI IDPs. Purple indicates the sex-specific DTI IDPs. Green indicates the sex-specific sMRI IDPs. **B** The sex-specific brain volume that more associated in females. **C**, **D** The sex-specific white matter tracts, **D** blue indicates the sex-specific white matter tracts of males. *scz* schizophrenia.

For UKB, 44 IDPs showed statistically significant genetic associations with SCZ PRSs. The SCZ PRSs were significantly correlated with one weighted mean MD in tract corticospinal tract right (Fig. 3D) and nine independent components derived from resting-state fMRI in males (Table S4), while females showed significant associations with 39 IDPs including one brain volume (Fig. 3B), 13 FA (Fig. 3C, D), and 25 rsfMRI components (Table S4). Five rsfMRI components were significantly correlated with SCZ PRSs in both males and females. Four of the five components showed significantly stronger correlations in males than females (FDR_bh_ = 1.15 × 10^−226^–5.84 × 10^−75^, *r* = 0.58–1.0). Independent component191 was the only component in the five that had a stronger correlation in females (FDR_bh_ = 2.24 × 10^−124^, *r* = 0.75) (Table S4).

The sex differences were also observed for BD and MDD PRSs in UKB. PRSs of both BD and MDD showed stronger genetic effects on brain functions in females than males. The effects of BD PRSs were significant on 11 white matter abnormalities and three rsfMRI components only in females. The effects of MDD PRSs were significant on 23 DTI regions and three rsfMRI ICA components also only in females (Fig. 3A and Table S4).

We replicated our sex-specific IDP results in a replication cohort. Of the 44 IDPs that were significantly associated with PRSs of SCZ identified in the discovery UKB analysis, 27 (61.4%, *p* = 0.048, Binomial test) had the same direction of sex differences in the replication study (Table S7).

### Mediation of IDPs on the genetic associations between PRSs of SCZ and cognition

Based on the analyses above, we found sex differences in the effects of SCZ PRSs on most cognitive functions in children. However, for adults, the effects of SCZ PRSs on fluid intelligence were not significantly different between the sexes. We performed a mediation analysis to investigate possible causes of these inconsistent results for UKB data. We focused on the mediation effects of the 44 IDPs in UKB, which were identified to be significantly associated with SCZ PRSs. The direct effect estimates of SCZ PRSs on fluid intelligence were consistently significant in the negative direction and stronger in females than males in the analyses for all the 44 IDPs (FDR_bh_ = 1.80 × 10^−54^–1.66 × 10^−8^, *r* = 0.12–0.35). This indicates the heterogeneity of the pathway from SCZ PRSs to cognition between males and females.

Although the marginal effect of SCZ PRSs on cognition did not differ significantly, the mediation effects of the related IDPs were different. The related IDPs could be divided into two types: (1) The indirect effects of SCZ PRSs on cognition through IDPs were negative for both sexes with males stronger than females; (2) The indirect effects of SCZ PRSs on cognition through IDPs were positive in females while nonsignificant or negative in males. We defined these related IDPs as type1 and type2, respectively, in what follows. The heterogeneity of the direct effects and the mediation effects of the related IDPs could lead to the homogeneous marginal effects of SCZ PRSs on cognition. We identified weighted mean MD in tract corticospinal, Mean FA in cingulum cingulate gyrus, and ICA component 149 as the type1 IDPs. The mediation effects were not significant in females while significantly negative in males for these IDPs. The type2 IDPs included ICA component 11 and ICA component 69. Contrarily, the mediation effects were not significant in males while significantly positive in females for type2 IDPs. The detailed results on the mediation effects of all the 44 IDPs were summarized in Table 3.

**Table 3:** Different types of effects present in mediation analysis.

*ab*		*c*′		*R*^2^(*ab*)	*R*^2^(*c*′)		Numbers
M	F	M	F				
Zero	Zero	–	–		F > M	Other mediators	20
Zero	–	–	–	F > M	F > M	Other mediators	13
–	–	–	–	F > M / NS	F > M	Other mediators	4
–	Zero	–	–	M > F	F > M	Cancel out	3
Zero	+	–	–	F > M	F > M	Cancel out	2
+	+	–	–	NS	F > M	Other mediators / Cancel out	2

*ab* the third variable effect; *c*′ direct effect; R^2^*(ab)* the sex difference of the partial R^2^ of the indirect effect of SCZ PRS on cognitive function; R^2^*(c*′*)* the sex difference of the partial R^2^ of the direct effect of SCZ PRS on cognition. *Numbers* the number of different conditions in the mediation analysis. *F* female; *M* male; − negative effects; + positive effects; *Zero*, *NS* nonsignificant. Significance was set at FDR < 0.05.

## Discussion

In this paper, we studied the heterogeneity of sex in the genetic associations between PRSs of six psychiatric disorders and related phenotypes in children and adults. The sex-specific results based on genetically derived disease risk scores are more powerful and comprehensive than those based on epidemiologically observed sex differences, which are generally affected by cultural, geographical region, and other environmental factors. Up to the present, diagnosis of psychiatric disorders has been based on behavioral presentation. Although a set of core characteristics of complex psychiatric disorders can be identified by clinicians, there are still controversies about diagnostic criteria and inconsistently used by exports [22]. Genetic-based analyses can more accurately uncover disease mechanisms, characterize individual differences, and resolve the differences in the literature.

We first observed the opposite relationship of the cognitive performance of females versus males in children and adults. After adjusted by age and other potential confounds that are relevant to neurocognitive (Supplementary Materials), the cognitive scores of girls were statistically significantly higher than boys in children while females were significantly lower than males in adults. To confirm that the phenotypic heterogeneity was not driven by the different cognitive tests applied, we used *g* factor as the general measurement of cognition for children in ABCD (Supplementary Materials) [38]. These results indicate that sex differences in cognitive functions change with development. Males show relatively late development in cognition.

We later investigated the effects of PRSs of psychiatric disorders on cognition and behaviors. Biased sampling in genetic studies can lead to spurious sex-specific effects [49]. We also investigated potential ascertainment bias in our study (Supplementary Materials). The results showed that participation bias only had a negligible impact on our results. As previously described, ASD-related symptoms would emerge after the typical onset age, no significant evidence supporting ASD is rare in adults [22]. In this study, there exists obvious heterogeneity among psychiatric disorders. ASD and MDD were more associated with CBCL behaviors than SCZ, BD, BD1, and BD2. What’s more, different from other psychiatric disorders, the PRSs of ASD were significantly positively correlated with fluid intelligence, which is consistent with the previous study [50]. As a comparison, cognitive function is a core feature of SCZ. Of the six psychiatric disorders, the PRSs of SCZ showed the most pervasive associations with cognition, which were only seen in boys. However, in adults, the PRSs of SCZ are significantly associated with the fluid intelligence of both sexes without significant sex differences. This inconsistency of sex difference could be due to differences in brain development or the effects of mediation on brain structure and function.

The human brain is responsible for cognition, behavior, and the regulation and destruction of brain development leading to psychiatric disorders. To study the mediation effect of the human brain on the pathway between cognition and genetic risk of psychiatric orders and its sexual heterogeneity, we examined the effects of PRSs of psychiatric disorders on brain structure and function. 38 sex-specific single IDPs were identified to be associated with the PRSs of SCZ, ASD, and MDD in adults. Most of these IDPs have been reported to be associated with psychiatric disorders in the literature (Table S4), e.g., cingulum cingulate gyrus. However, due to the limited sample size and varying brain development at different stages, the correlation between brain imaging traits and PRSs of psychiatric disorders was not significant in children.

Sex differences were identified in the mediation function of IDPs. The sex differences in the effects of SCZ PRSs on cognition were significant in children while absent in adults. The sex differences in the mediation function of IDPs could be the cause. Our results of the mediation analysis showed that the direct effects of SCZ PRSs on the fluid intelligence of females were stronger than those of males. Five IDPs including weighted mean MD in tract corticospinal, Mean FA in cingulum cingulate gyrus, ICA component 149, ICA component 11, and ICA component 69 showed compatible sex differences in mediation effects with marginal effects, which can explain the inconsistency of sex differences in the effects of SCZ PRSs on cognition between children and adults. The identified IDPs highlight the sexual heterogeneity in their mediation effects on the developmental mechanism between SCZ and cognition.

Our work brings about multiple potential applications. First, our findings can improve the guidelines for screening of SCZ and their effectiveness by establishing characterized screening ages for individuals with different genetic risks. For example, we have shown that the negative correlation between cognition and SCZ PRSs is more significant in boys. Differentiated strategies can be applied to boys and girls in the population screening for SCZ. We can recommend that boys at risk for SCZ begin their screening at age 9–10. We can perform routine monitoring on the elderly and suggest more focus on females’ cognitive decline as cognitions in females were more strongly associated with SCZ PRSs. Second, the sex-specific correlations between PRSs of psychiatric disorders and brain imaging in different age groups can improve our understanding of the roles that the brain regions play in the development of sex-biased psychiatric disorders.

This study has the following limitations. Our results warrant replications in patients. So far, we only performed our analyses on healthy individuals in UKB and ABCD. In other words, the PRSs were calculated only for healthy individuals due to a lack of access to diseased subjects. The replication based on individual-level data of patients could provide powerful evidence for the identified sex differences in the correlation between genetic risk of psychiatric disorders and related traits. The cross-sectional nature of the data analyzed from the ABCD and UKBB subjects is also a limitation. In view of the significant heterogeneity between adults and children of sex differences in genetic associations between cognitive functions and psychiatric disorders, the heterogeneity might be attributed to the relative developmental timing of different brain regions. A large-scale longitudinal dataset is still desirable for causal inference on genetic risk and related phenotypes.

In conclusion, late childhood is an important period for sexes to differentiate their psychiatric risk. The genetic risk of SCZ demonstrated more correlation with cognitive functions in boys than in girls. The dynamic sex differences in cognitive functions, as well as the genetic correlations between psychiatric disorders risks and cognitive functions, may partly owe to the sex differences in brain development. We found twenty brain regions showed differences between sexes in mediation effects from psychiatric disorders risks to cognitive functions. Our findings help pinpoint the underlying early etiology of psychiatric diseases, reveal sex differences in the effects of genetic risk of psychiatric disorders, and improve screening and precaution of psychiatric disorders.

## Supplementary information

Supplemental material

TableS1-7

## Data Availability

We made use of publicly available software and tools. All codes used to generate results that are reported in this paper are available upon request.
