# Supplementary material for: Sex-specific genetic association between psychiatric disorders and cognition, behavior and brain imaging in children and adults
Source: Transl Psychiatry. 2022 Aug 26;12:347. doi: 10.1038/s41398-022-02041-6 (PMC9418275; doi:10.1038/s41398-022-02041-6)
Supplement: Supplementary file 1 — Supplemental material [file 41398_2022_2041_MOESM1_ESM.docx]

**Supplementary Information**

**Sex-specific genetic association between psychiatric disorders and cognition, behavior and brain imaging in children and adults**

**Demographics and representativeness.**

**UKB**: We compared the sexes on socioeconomic status that may be relevant to neurocognitive phenotypes. Females have higher neighborhood deprivation level scores (data field #189) than males (Wilcoxon test *p*=0.0029, effect size(r)=0.0078). Females reported a significantly lower household income (data field #738) (chi-square test p < 2.2e-16, Cramer’s V = 0.034). Notably, these two measures were for neighborhood and household, respectively, so they contained additional noise compared to personal measures. There no personal occupation or income measure was available in the UKB.

Next, we compared educational attainment (data field #6138), one of the personal measures of socioeconomic status. Subjects were removed based on the answer “None of the above” and “Prefer not to answer”. 41.6% (28,756 / 69,159) females and 45.5% (27,382 / 60,269) males reported having college/university degree. Although in general, the proportion of individuals has a college degree is higher. The proportion is about equal between the sexes for college degree.

**ABCD:** Parents reported their family income ranging from 1 to 10 (#pdem02). There was no significant difference between boys and girls for family income (chi-square test *p*=0.68).

For caregiver education, parents reported their years of schooling (#pdem02) ranging from 0 for no formal education to 21 doctoral degrees. We compared parental educational attainment with no sex difference (chi-square test *p*=0.16). 82.02% (1,724 / 2,102) parents for girls and 81.97% (1,944 / 2,401) parents for boys reported having college/university degree (completed their full-time education >= 16).

**Age.** The mean age of females was ($57.46\pm8.08$) years, and the mean age of males was ($58.39\pm8.36$) years. Males were statistically significantly older than females (Wilcoxon test, *p* < 2.2e-16, r *=* 0.11).

The mean age of girls was ($9.89\pm0.61$) years, and the mean age of boys was (9.92$\pm0.62$) years. There was no statistically significant difference between sexes (Wilcoxon test *p* = 0.14).

**Genotype data.** The genotype data for ABCD comprised 10,627 samples and were released in 2019. SNPs and subjects were excluded based on the following quality control criteria: SNP not in Hardy-Weinberg disequilibrium (*P* < ${10}^{-10}$), with > 5% missing data, or minor allele frequency (MAF) < 5%. Samples were excluded if there was a mismatch between genetic sex and self-reported sex or genotype missingness > 5%. We used PLINK v1.9^1^ to identify a set of pruned autosomal variants to estimate heterozygosity and genomic relatedness, then removed samples with extreme heterozygosity (> ±3 SD), and deleted one individual from each related pair with more missingness variants. We further merged ABCD data with the 1000 Genomes Project dataset and performed admixture^2^ analysis to identify individuals of European ancestry. SHAPEIT^3^ and IMPUTE2^4^ were used to phase and impute the 5,735 individuals of European ancestry using the 1000 Genomes Project phase 3^5^ imputation reference panel. Stringent quality control was applied as described above after imputation. Principal component analysis (PCA) was performed using PLINK v1.9 to remove genetic outliers. Recalculated top three principal components (PCs) were included as covariates.

**Sex-differential participation bias**

As noted in Paristu et al^6^, biased sampling in genetic studies can lead to spurious sex-specific effects. To assess whether our study was impacted by such ascertainment bias, we performed GWAS for sex in 4 different cohorts in our paper including ABCD, UKB fluid score cohort, UKB brain imaging discovery cohort, and UKB brain imaging replication cohort. Age and PCs of genotype were set as independent variables. None of the four GWAS identified significant signals (Fig. S2). In addition, the 158 independent GWAS signals identified in Paristu et al. were enriched in none of the top signal lists of the four GWAS.

We also calculated the percentages of the samples whose phenotypes were above median for males and females for the phenotypes included in the study. Most of the percentages were around 50% in ABCD and UKB fluid score cohort (41%-58%). Only 57of the 519 IDPs in UKB brain imaging discovery cohort, 60 of the 519 IDPs in UKB brain imaging replication cohort, and 128 of the 1151 brain measures in ABCD cohort had males above the median more than 61%. Please note that sex differences exist in the human brain^7^. These results showed that participation bias only had a negligible impact on our results.

**Polygenic risk score**

As noted in Ding et al^8^, the PRSs constructed using GWAS summary statistics involve uncertainty. The standard association tests based on linear regression may be questionable due to the PRS uncertainty^9^. We evaluated association between the constructed PRS and a number of phenotypes. However, the concept of uncertainty actually differs in these two papers. Ding et al. focused on the inferential variance of PRS caused by the Markov Chain Monte Carlo (MCMC) algorithm. They showed that compared with the cohort level summary of correlation coefficient, variance of individual PRS is high especially for those in the top percentile. However, in our analysis, we focused on the variance explained by PRS at the cohort level and did not study the PRS-based ranking of individuals.

Wang et al.^9^ focused on correcting the anti-conservative bias of using predicted outcomes in the regression model as their variance is usually smaller. The application of Wang's method requires the information of individual-level disease status y and the corresponding predicted PRS y_p on the testing dataset to learn the simple relationship between a phenotype and PRS, k (y, y_p) for correction. However, the individual-level information of disease status is not available in our datasets. The diagnosis of autism and schizophrenia and ABCD 2.0.1 is not available so far. Children with moderate to severe neurological issues were ruled out from ABCD2 to eliminate the influence of the disorders on the downstream testing of the study. There were very few kids who met full criteria of bipolar and major depressive disorders. Also, there are very few samples who have one of the six psychiatric disorders in UKB brain imaging data (according to ICD-10: SCZ 12; BD 53; MDD 698; ASD 0).

We would also like to point out that we did not use a simple linear regression to test the association between phenotypes and PRS. Instead, we focused on testing the difference of interpretability (R-square) of PRS on phenotype between males and females.

**Statistical analysis**

**Adjustment:** Two similar but distinct approaches were performed to estimate whether the potential confounds relevant to cognitive functions such as socioeconomic status affect the differences between sexes in UKB healthy subsample. Firstly, we used linear regression to adjust neighborhood deprivation level, household income, educational attainment, and age using cognitions as the dependent variable, then compared the residuals. In the propensity score matching^10^ analysis, the females and males with similar distribution of these confounders were matched into two subgroups. For both comparisons, we consistently found that the average fluid scores were higher in males relative to females (Wilcoxon test $p=1.15\times{10}^{-22}$ r = 0.036 and $p=8.06\times{10}^{-22}$, r = 0.036).

After adjusted by age, family income, and parents education, most cognitive scores of girls were higher than boys from ABCD (Wilcoxon test: card sort, ${FDR}_{bh}=1.65\times{10}^{-5}$, r=0.067; pattern, $FDR_{bh}=1.43\times{10}^{-7}$, r=0.084; picture sequence, $FDR_{bh}=1.43\times{10}^{-7}$, r=0.083; fluid, ${FDR}_{bh}=9.20\times{10}^{-6}$, r=0.07; total cognition, ${FDR}_{bh}=0.0080$, r =0.042).

**Multiple comparison adjustments:**

**Cognitive functions:** In cognitions and psychiatric behaviors analysis of ABCD study, we performed permutation test to evaluate the statistical significance of the observed partial R^2^ of the regression for each sex. Specifically, we permute 10,000 times to produce 10,000 random R^2^s for each sex, then we saved the number of random R^2^ above the observed R^2^. The ratio of the number above observed R^2^ to 10,000 is the estimated p-value. Then the Benjamini-Hochberg Procedure^11^ that controls the false discovery rate (FDR) was applied to ﻿address the multiple comparison problem.

**Brain imaging:** For the brain imaging analysis both in ABCD and UKB study, based on the distribution of the partial R^2^ from the 10,000 permutations, we made multiple comparison adjustments using the false discovery rate (FDR) to assess the statistical significance of the observed partial R^2^. For each permutation, there were a total of 1,150 and 519 R-square values in ABCD and UKB, respectively. We considered a specific threshold, then saved the number of R-square above that threshold. We took the average number of these 10,000 numbers, that gives an estimated number of false positives above the threshold. Next, we calculated the number of R-square above the threshold for the observed data. The ratio of the average number from 10,000 permutations to the number from the observed data is the estimated false discovery rate (<0.05). In this method, the relations between different brain regions were considered.

We use the following Fig. S3 to illustrate our pipeline of the statistical analyses in this study.

**Mediation analysis:**

Mediation analysis was carried out to investigate whether sex-specific IDPs mediate the link between SCZ PRSs and cognitive function, and their heterogeneity between the sexes. The single model is shown in Fig. S4 and the following Equations 1 to 3:

$Y=\beta_{1}+cX+e_{1},$ (1)

$Y=\beta_{2}+c^{'}X+bM+e_{2},$ (2)

$M=\beta_{3}+aX+e_{3}$. (3)

where *Y* is the measured cognitive functions, *X* is the SCZ PRSs, and *M* is the sex-specific ROI. The $\beta_{1},\beta_{2},\beta_{3}$are intercepts, and $e_{1},e_{2},e_{3}$ are residuals. In Equation 1, the coefficient *c* represents the total effect of *X* on *Y*. In Equation 2, the c’ represent the links *X* to *Y* controlling for *M*. The coefficient b represents the links between M and *Y* controlling for *X*. In Equation 3, the coefficient *a* indicates the relation between *X* and *M*. We used percentile bootstrap confidence intervals method^12, 13^ to test the significance of mediated effect ($\hat{ab}$).

The statistical significance test for the sex difference of direct and indirect effects was conducted in two steps: First, we calculated the indirect and direct R-square based on Equation 2. Next, we used the Wilcoxon test to compare the 10,000 R-squares (bootstrapped 10,000 iterations) between sexes, respectively.

**General cognitive function (g):** A number of cognitive tests have been used to assess different cognitive domains in each cohort. In order to compare these cognitive tests uniformly, g factors, a latent trait underlying shared variance across multiple subdomains of cognition^14^ was conducted, which previously showed a high genetic correlation with fluid test from UKB^15^. We extracted g factors from seven tests (including Picture Vocabulary, Flanker, List Sorting Working Memory, Card Sort, Pattern Comparison Processing Speed, Picture Sequence Memory, Reading Recognition) of NIH Toolbox for ABCD using principal component analysis (PCA). The first principal component obtained accounted for 40.04% of the variance in overall test performance. For healthy children in the ABCD study, girls showed higher g factor than boys (t-test $p=9.84 \times{10}^{-7}$, Cohen's d = 0.25)

**Fig. S1.** Workflow of the quality control for Adolescent Brain Cognitive Development (ABCD) and brain imaging of UK Biobank (UKB).

**Fig. S2.** Manhattan plot of genetic variants associated with sex in four cohorts.

**Fig. S3.** The pipeline of the statistical analyses in this study.

**Fig. S4.** Mediation model.

**Table S1.** Measures for this study.

**Table S2.** The effects of PRSs for six psychiatric disorders on cognitive functions and psychiatric behaviors in the ABCD dataset.

**Table S3.** The effects of PRSs for six psychiatric disorders on fluid intelligence in UK Biobank dataset.

**Table S4.** The sex-specific imaging-derived phenotypes (IDPs) were associated with psychiatric disorders in the UK Biobank dataset.

**Table S5.** The brain ROI overlaps between ABCD and UKBB.

**Table S6.** Mediation analysis.

**Table S7.** The R-square and rank ratio of 44 sex-specific IDPs in of the discovery and replication sets.

**Fig. S1.** **Workflow of the quality control for Adolescent Brain Cognitive Development (ABCD) and brain imaging of UK Biobank (UKB).** **a** The first stage of the pipeline is to perform pre-imputation quality control of the data. Then performed admixture analysis to select European ancestry participants. The following step was to phase and impute data set. Stringent quality control was applied as described in the first stage after imputation. Principal component analysis (PCA) was performed and removed genetic outliers according to PC1 and PC2. **b** The first step was to select European participants according to UKB ID 22006, then match the genotype with the phenotype. To perform quality control. Close relatives (estimated kinship coefficient > 0.0442) were removed. Selecting the European individuals who no genetic kinship to other participants (#22021) as the discovery dataset, and the remaining unrelated individuals as a replication dataset.

**Fig. S2. Manhattan plot of genetic variants associated with sex in four cohorts.**

Each point reports a genetic variant. The red dotted line indicates the statistical significance threshold ($P<5\times{10}^{-8}$). The blue points indicate variants included in 158 significant loci identified by Paristu et al.


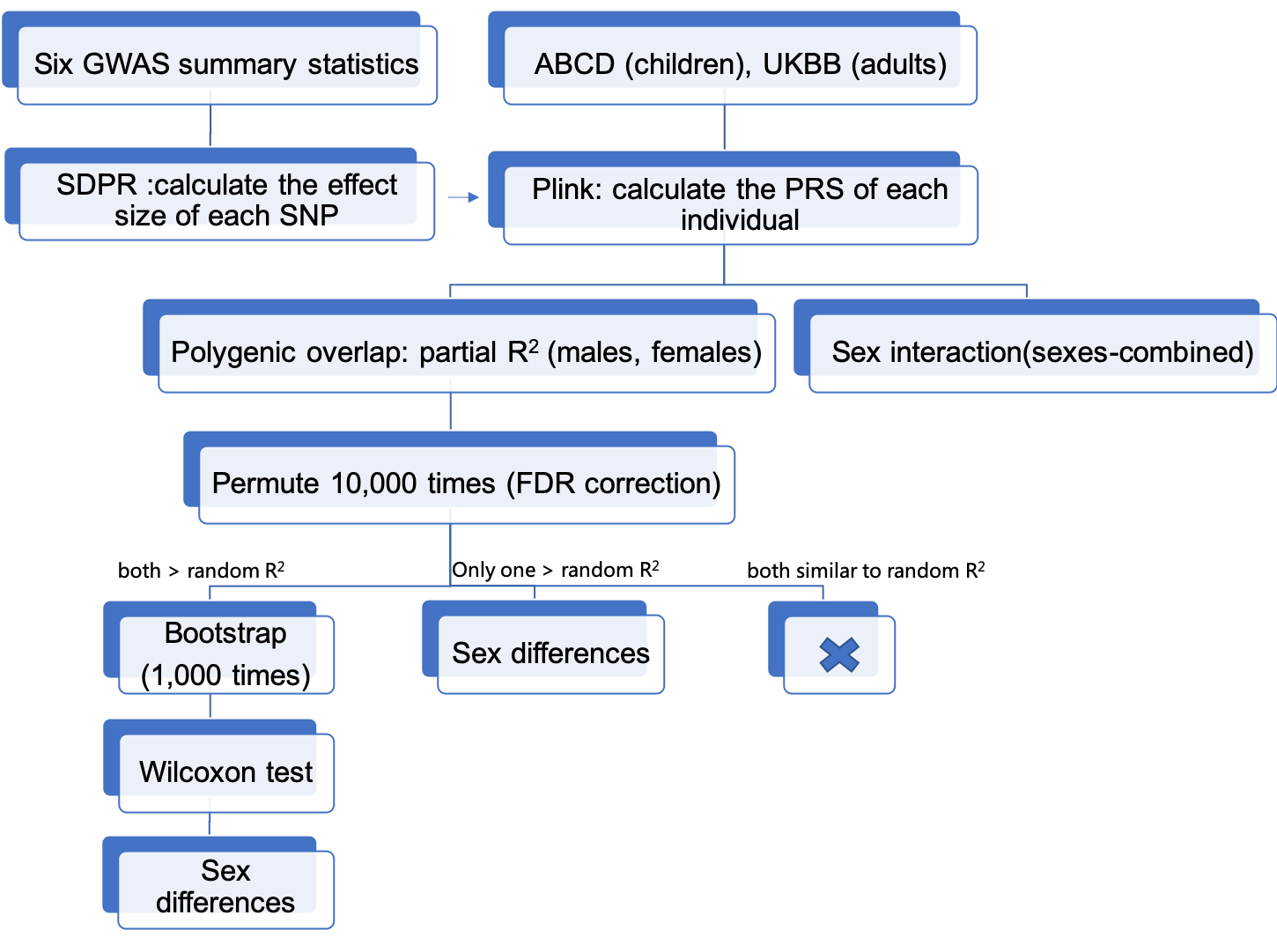


**Fig. S3.** The pipeline of the statistical analyses in this study.

**Fig. S4. Mediation model.** In this framework, the relationship between SCZ PRSs and cognition is decomposed into two pathways: one links SCZ PRSs to cognition directly(c’), and the other links SCZ PRSs to cognition through sex-specific ROI (ab). c represents the total effect of SCZ PRSs on cognition.

**Supplemental References**

1. Purcell S, Neale B, Todd-Brown K, Thomas L, Ferreira MA, Bender D *et al.* PLINK: a tool set for whole-genome association and population-based linkage analyses. *Am J Hum Genet* 2007; **81**(3)**:** 559-575.

2. Alexander DH, Novembre J, Lange K. Fast model-based estimation of ancestry in unrelated individuals. *Genome Res* 2009; **19**(9)**:** 1655-1664.

3. Delaneau O, Marchini J, Zagury JF. A linear complexity phasing method for thousands of genomes. *Nat Methods* 2011; **9**(2)**:** 179-181.

4. Howie B, Marchini J, Stephens M. Genotype imputation with thousands of genomes. *G3 (Bethesda)* 2011; **1**(6)**:** 457-470.

5. Genomes Project C, Auton A, Brooks LD, Durbin RM, Garrison EP, Kang HM *et al.* A global reference for human genetic variation. *Nature* 2015; **526**(7571)**:** 68-74.

6. Pirastu N, Cordioli M, Nandakumar P, Mignogna G, Abdellaoui A, Hollis B *et al.* Genetic analyses identify widespread sex-differential participation bias. *Nat Genet* 2021; **53**(5)**:** 663-671.

7. Ritchie SJ, Cox SR, Shen X, Lombardo MV, Reus LM, Alloza C *et al.* Sex Differences in the Adult Human Brain: Evidence from 5216 UK Biobank Participants. *Cereb Cortex* 2018; **28**(8)**:** 2959-2975.

8. Ding Y, Hou K, Burch KS, Lapinska S, Prive F, Vilhjalmsson B *et al.* Large uncertainty in individual polygenic risk score estimation impacts PRS-based risk stratification. *Nat Genet* 2022; **54**(1)**:** 30-39.

9. Wang S, McCormick TH, Leek JT. Methods for correcting inference based on outcomes predicted by machine learning. *Proc Natl Acad Sci U S A* 2020; **117**(48)**:** 30266-30275.

10. PAUL, R., ROSENBAUM, DONALD, B., RUBIN. The central role of the propensity score in observational studies for causal effects. *Biometrika* 1983; **70**(1)**:** 41-55.

11. Benjamini Y, Hochberg Y. Controlling the False Discovery Rate: A Practical and Powerful Approach to Multiple Testing. *Journal of the Royal Statistical Society Series B: Methodological* 1995; **57**(1)**:** 289-300.

12. Hayes A. Introduction to mediation, moderation, and conditional process analysis. *Journal of Educational Measurement* 2013; **51**(3)**:** 335-337.

13. Preacher KJ, Hayes AF. SPSS and SAS procedures for estimating indirect effects in simple mediation models. *Behavior Research Methods Instruments & Computers* 2004; **36**(4)**:** 717-731.

14. Carroll JB. Human cognitive abilities: A survey of factor-analytic studies. *Acta Psychologica* 1995; **88**(3)**:** 261-264.

15. Davies G, Lam M, Harris SE, Trampush JW, Luciano M, Hill WD *et al.* Study of 300,486 individuals identifies 148 independent genetic loci influencing general cognitive function. *Nat Commun* 2018; **9**(1)**:** 2098.
